# Evolutionary ecology of host competence after a chytrid outbreak in a naive amphibian community

**DOI:** 10.1098/rstb.2022.0130

**Published:** 2023-07-31

**Authors:** Ana V. Longo, Karen R. Lips, Kelly R. Zamudio

**Affiliations:** ^1^ Department of Biology, University of Florida, Gainesville, FL 32611, USA; ^2^ Department of Biology, University of Maryland, College Park, MD 20742, USA; ^3^ Department of Integrative Biology, College of Natural Sciences, The University of Texas, Austin, TX 78712, USA

**Keywords:** community-level disease dynamics, key hosts, species traits

## Abstract

Naive multi-host communities include species that may differentially maintain, transmit and amplify novel pathogens; therefore, we expect species to fill distinct roles during infectious disease emergence. Characterizing these roles in wildlife communities is challenging because most disease emergence events are unpredictable. Here, we used field-collected data to investigate how species-specific attributes influenced the degree of exposure, probability of infection, and pathogen intensity, during the emergence of the fungal pathogen *Batrachochytrium dendrobatidis* (*Bd*) in a highly diverse tropical amphibian community. Our findings confirmed that ecological traits commonly evaluated as correlates of decline were positively associated with infection prevalence and intensity at the species level during the outbreak. We identified key hosts that disproportionally contributed to transmission dynamics in this community and found a signature of phylogenetic history in disease responses associated with increased pathogen exposure via shared life-history traits. Our findings establish a framework that could be applied in conservation efforts to identify key species driving disease dynamics under enzootics before reintroducing amphibians back into their original communities. Reintroductions of supersensitive hosts that are unable to overcome infections will limit the success of conservation programmes by amplifying the disease at the community level.

This article is part of the theme issue ‘Amphibian immunity: stress, disease and ecoimmunology’.

## 1. Introduction

The emergence of highly virulent multi-host pathogens in sea stars, amphibians, snakes and bats emphasizes the importance of identifying ecological and evolutionary processes influencing transmission across host species [1–5]. Multi-host pathogens cause unprecedented biodiversity loss [6,7], therefore quantifying the distinct roles that species fill during the emergence of infectious disease can improve our understanding of the outcome of outbreaks [8–10]. Theoretical models investigating parasites in communities provide a starting point to link within and among host responses [11]. Unfortunately, because most disease emergence events are unpredictable, we have few long-term datasets on host ecology and pathogen surveillance that span epizootic outbreaks, precluding the empirical measurement of the contributions of naive species to community-level disease dynamics.

Here, we focused on a unique epidemiological dataset obtained during an outbreak of chytridiomycosis, caused by the fungus *Batrachochytrium dendrobatidis* (*Bd*), at a species-rich cloud forest amphibian community at El Copé, central Panamá [1]. Previous studies at this site have demonstrated that: (i) *Bd* was absent before the year 2004 [1,12]; (ii) *Bd* emergence caused major declines in species richness and population densities [1,13], thus impacting host evolutionary history and community structure [1,13–15] and their predators [7]; (iii) amphibians seem to be persisting with the fungus [16,17]; and (iv) amphibian declines resulted in increased incidence of mosquito-borne disease in humans [18]. The pre-epizootic host community included 74 species of amphibians [1,12,14] of which 30 (41% of total amphibian diversity) disappeared following the emergence of *Bd* in 2004 [14]. Naive and highly diverse communities, such as the El Copé amphibian assemblage, probably consisted of a continuum of host types, ranging from those that increase community vulnerability to pathogens, to those that contribute to community persistence [11,19]. Therefore, this dataset permits us to expand from studies of single species host–pathogen dynamics [20,21] to multispecies assemblages by quantifying species-specific contributions at various stages of the epizootic event. In addition, conservation strategies post-epizootics require methods to predict how reintroducing different amphibian species (especially those that are still susceptible to infection) may affect community-level disease dynamics [22].

Pathogen growth models from single-species hosts predict that the rapid increase of individuals with high-intensity infections leads to population extirpation [20,21], whereas persistence is characterized by the survival of individuals with low-level infections [20]. We applied these predictions to amphibians at El Copé to test whether the persistence of species in multi-host assemblages relates to infection levels [23]. High ecological and evolutionary diversity among species within this tropical community [14] should have created different opportunities for pathogen exposure and proliferation. For instance, species with a high probability of decline owing to *Bd* should show similar characteristics including high infection intensities, high contact rates, and use of habitats that support pathogen survival or transmission [24]. By contrast, less susceptible hosts will carry low infection intensities, have fewer contacts, or spend less time in pathogen hotspots, leading to their persistence after the outbreak. If these traits have an evolutionary component that links species to pathogen exposure or susceptibility [9,25,26], we expected to find a phylogenetic signal for infection responses and survival across the community. Therefore, by quantifying community composition and disease responses during the outbreak, we can partition species contributions and determine whether they exacerbate or limit community-wide infection dynamics [27].

In this paper, we estimated persistence based on a model of species-specific traits from communities of Central American amphibians that experienced declines attributed to chytridiomycosis epizootics [1,12,28]. This model was applied to the intact community of El Copé, to generate for each species, a probability of decline owing to chytridiomycosis based on distributional and ecological characteristics [12]. We quantified temporal changes in infection for all species in the community during and after the epizootic at El Copé. For each species, we averaged infection intensity for individuals present during the four-month outbreak; this measure represents each species' first response given innate immunity, species’ traits, and environmental factors influencing susceptibility. To determine whether evolutionary history mediated infection responses during the outbreak, we tested for a phylogenetic signal in prevalence, infection intensity, and persistence probability. We expected that host reproductive mode would be an important trait for pathogen exposure and transmission across species [29], because it directly links hosts with presumed pathogen habitats [30]. Thus, we also tested for phylogenetic signal in disease responses by separating species by their reproductive mode. Finally, we determined the role of each species in community-wide infection dynamics by partitioning their contribution in terms of their prevalence, abundance and infectiousness [8]. Although our data came from a natural experiment in the wild, our framework can be applicable to naive communities by experimentally testing each species susceptibility and quantifying species abundance. In addition, this approach expands the tools to study the emergence of new strains or pathogens in diverse animal communities.

## 2. Methods

### (a) Linking host traits and infection parameters

We analysed a total of 898 DNA extracts from individual swabs before and during the epizootic [1]. In addition, we quantified *Bd* from 89 individuals from the enzootic stage in 2008 and 2010 (electronic supplementary material, table S1). For additional information about field sampling please refer to the electronic supplementary material. We refer to *Bd* prevalence as the number of *Bd* positive individuals over the total sampled for each species at a particular time point. We measured infection intensity as the mean number of *Bd* zoospore genomic equivalents (GE > 0) detected on each individual. Average infection intensity was estimated for 45 species sampled at El Copé during and after the epizootic (electronic supplementary material, table S1). We did not remove uninfected individuals to calculate average infection levels. We deposited the code and data on https://github.com/anavlongo/evoeco_hostcompetence.

We transformed species-specific decline probabilities into trait-based persistence probabilities (1 – decline probability obtained from Lips *et al*. [12]). Lips *et al*. [12] predicted the probability of decline for each species at El Copé using distributional and ecological traits, as well as population responses to chytridiomycosis of other amphibian species from multiple upland sites in Costa Rica and Panama (refer to Appendix 1 in Lips *et al*. [12]). Considering the spatial pattern of disease spread across Central America [28], we assume that decline probabilities probably represent population declines owing to *Bd*. However, the distributional and ecological traits considered in Lips *et al*.'s model are also known to increase extinction risk owing to other threats such as habitat loss and climate change [29,31,32]. We confirmed the fit of estimated trait-based persistence with observed survival using a logistic regression in *R* [33]. Subsequent linear regression analyses included only species with complete records for each of the parameters (persistence, prevalence, infection intensity), and that were present in the community phylogeny (total of 37 species, see the electronic supplementary material, table S1).

We performed univariate linear regressions between persistence probability, disease prevalence and average log-transformed infection intensity for each species [33]. We used the R package *gvlma* to perform a global validation of all linear model assumptions and to evaluate skewness, kurtosis and heteroscedasticity [34], and found that all variables met the assumptions of linear regressions. Regression diagnostics identified five species as potential outliers (*Craugastor bransfordii*, *Craugastor crassidigitus*, *Craugastor punctariolus*, *Espadarana prosoblepon* and *Pristimantis cruentus*). We excluded *C. bransfordii* and *C. punctariolus* [35] from further analyses because these species declined almost immediately after *Bd* arrival and we did not have sufficient swab samples for downstream analyses.

### (b) Testing for phylogenetic signal in host responses

**Figure 1.**
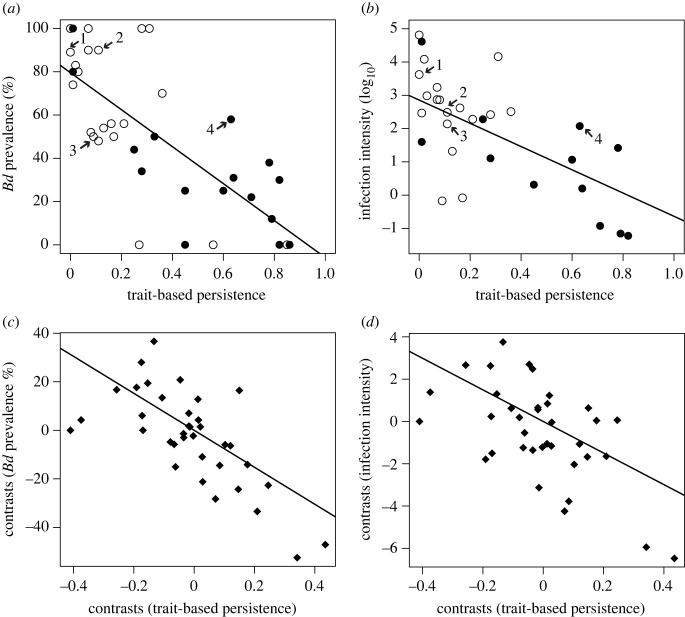
Species-specific predicted persistence and disease responses for 37 amphibian species at El Copé, Panamá during the 2004 *Bd* outbreak. (*a*,*b*) Trait-based persistence relative to prevalence and infection intensity differed in species with aquatic larvae (open circles) and terrestrial breeding (closed circles). Indicator species associated with epizootic are numbered: (1) *Atelopus varius*, (2) *Silverstoneia nubicola*, (3) *Espadarana prosoblepon* and (4) *Pristimantis cruentus*. (*c*,*d*) The same patterns were maintained in regressions using phylogenetically independent contrasts.

We assessed phylogenetic signal in species responses to disease (persistence probability, prevalence and infection intensity) by calculating Blomberg's *K* based on 1000 randomizations (electronic supplementary material, table S3) using the package *picante* and *phytools* in R [36–38] and using the phylogenetic tree for the El Copé amphibian community [14]. We accounted for intraspecific variability in infection intensity (electronic supplementary material, table S3) by incorporating estimation error with pooled variance with the package *phytools* in *R* [38,39]. To control for the effect of evolutionary similarity in disease dynamics among related species (figure 1), we repeated the linear regressions using phylogenetic independent contrasts (PICs) for each attribute (i.e. persistence, prevalence, infection intensity) calculated using the packages *ape* and *geiger* in R [40,41]. We ran simple linear regressions with resulting PICs (figure 1*c,d*) and evaluated the linear model assumptions with the package *gvlma* [34].

We further tested whether individual-level infection intensities were related to taxonomic family membership using a Kruskal Wallis test in R (electronic supplementary material, table S2). Of the 12 families that comprised the pre-decline amphibian community, we selected the eight families with more than 10 individuals to permit resampling of data 10 000 times using the package *coin* in R [42].

### (c) Community-level analyses of key hosts

#### (i) Indicator species analysis

We identified the species that were associated with major changes in the community during the epizootic, by performing a multilevel pattern analysis [43]. We generated an amphibian community abundance matrix from the standardized transect surveys (*n* = 155 transects; [14]). For the indicator species analysis, we grouped surveys as a ‘site’ depending on its timing during the outbreak: pre-epizootic, epizootic, enzootic 2008, enzootic 2010 (*n*_pre_ = 15, *n*_epi_ = 119, *n*_2008_ = 7, *n*_2010_ = 14). We used the non-equalized point biserial correlation index (*r*_pb_) in the *R* package *indicspecies* [44] to evaluate the strength of the species associations to each phase of the epizootic event (electronic supplementary material, table S4). We defined as highly susceptible those species with significant association with the epizootic stage and high loads, because these species were highly abundant but disappeared after the outbreak.

#### (ii) Asymmetries in host heterogeneity

A species contribution to transmission dynamics (*π*) is comprised three separate measures: host abundance (*θ_A_* ), pathogen intensity (*θ_S_*) and pathogen prevalence (*θ_I_*) [8] (figure 3; data in the electronic supplementary material, table S5). We identified key hosts as those with the highest *π* [8]. For each host species, we calculated density $\left(\theta_{i}^{A}\right)$ from field survey data [14], prevalence $\left(\theta_{i}^{I}\right)$ and infection intensity $\left(\theta_{i}^{S}\right)$ from quantitative polymerase chain reaction of field swabs. We used infection intensity as a proxy for shedding rates because both Reeder *et al*. [45] and DiRenzo *et al*. [46] found a positive correlation between infection loads quantified from swabs and pathogen shedding rates.

## 3. Results

### (a) Species-specific traits dictate disease responses

We found that species-level variation in infection response was predicted both by the *a priori* estimate of trait-based survival probability, as well as observed survival after the epizootic (figure 1). Extirpated species carried a significantly higher *Bd* prevalence than species that persisted after the epizootic (*χ*^2^_1_ = 51.6, *p* < 0.001, *n* = 898; persisted prevalence: 48%; extirpated: 72%). Trait-based persistence probability was also negatively correlated with infection prevalence (*β* = −85.44 ± 12.88, *F_1, 35_* = 44.01, *p* < 0.001, *R*^2^ = 0.56; electronic supplementary material, table S1; figure 1*a*) and infection intensity (*β* = −3.49 ± 0.63, *F_1, 35_* = 30.67, *p* < 0.001, *R*^2^ = 0.47; figure 1*b*; electronic supplementary material, table S1). These patterns remained consistent even after we accounted for phylogeny using independent contrasts (prevalence: *β* = −76.19 ± 13.75, *F_1, 35_* = 30.73, *p* = 0.001, *R*^2^ = 0.47; figure 1*c*; infection intensity: *β* = −7.47 ± 1.85, *F_1, 35_* = 16.34, *p* < 0.001, *R*^2^ = 0.32; figure 1*d*).

### (b) Reproductive mode links species to pathogen exposure

**Figure 2.**
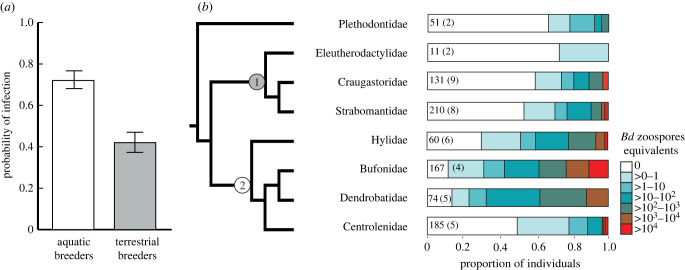
Impact of reproductive mode and phylogenetic history on *Bd* infection during the epizootic. (*a*) Individuals with aquatic larvae were four-fold more likely to be infected (odds ratio: 4.3, *z* = 9.7, *p* < 0.0001) than terrestrial breeders. (*b*) Infection intensity differed significantly among families (Kruskal Wallis: *H*_7_ = 202.8, *p* < 0.001) and development modes (Kruskal Wallis: *H*_1_ = 77.3, *p* < 0.001) during the 2004 *Bd* outbreak. We found a significant phylogenetic signal (*K* ≈ 1; electronic supplementary material, table S3) in prevalence only for individuals with aquatic larvae (clade 2). Colours represent pathogen loads (number of genomic equivalents of zoospores) by family; numbers represent total sample size and the number of species (in parentheses). (Online version in colour.)

Both family membership and reproductive mode were associated with the probability of infection and infection intensity (figure 2). The mean ranks of infection intensity significantly differed among families (Kruskal-Wallis Test: *H*_7_ = 202.8, *p* < 0.001; figure 2; electronic supplementary material, table S2), indicating potential limits on the pathogen's ability to reproduce within host species of certain families. Families Bufonidae, Centrolenidae, Dendrobatidae and Hylidae (aquatic breeders) carried higher pathogen burdens than families Craugastoridae, Strabomantidae, Eleutherodactylidae, Plethodontidae (terrestrial breeders) (figure 2; electronic supplementary material, tables S1 and S2). Indeed, individuals with aquatic larvae were four times more likely to be infected than terrestrial breeders (odds ratio test: 4.3, *Z* = 9.7, *p* < 0.0001; figure 2), confirming that reproductive mode is an important factor for exposure and susceptibility.

### (c) Phylogenetic correlates of disease responses

Using comparative methods, we detected a significant phylogenetic signal in average infection intensity (*K* = 0.61, *p* = 0.016; electronic supplementary material, table S3), confirming our family based and trait-based comparisons. Ignoring intraspecific variability in infection intensity resulted in lower *K* values (electronic supplementary material, table S3). We failed to detect a phylogenetic signal for prevalence across the entire community (*K* = 0.34, *p* = 0.08). An analysis of prevalence in individual clades detected significant *K* values only for aquatic breeders (*K* = 0.77, *p* = 0.045), showing that, in this clade, close relatives tend to share similar responses to the pathogen. The lack of significance in *K* for prevalence for the community resulted from high variation in prevalence among close relatives of terrestrial breeders. Likewise, we did not find a phylogenetic signal for persistence probability across the amphibian community (*K* = 0.38, *p* = 0.092), or after separating hosts into clades related to their reproductive mode.

### (d) Species associated with community change

Indicator species analysis identified seven species for which abundance was significantly associated with pre-decline or epizootic phases (electronic supplementary material, table S4). These species were identified as having low probability of survival, high prevalence and high infection intensity (electronic supplementary material, table S1), making them short-lived disease amplifiers [46]. Many of these species were extirpated from El Copé (e.g. *Atelopus varius* (formerly *zeteki*), *Craugastor evanesco, C. punctariolus, Silverstoneia nubicola, Hyalinobatrachium chirripoi, Colosthetus* spp*.*). By contrast, species significantly associated with later, enzootic phases generally had low *Bd* prevalence and infection intensity (even during the epizootic, electronic supplementary material, table S1), and high trait-based persistence probability; those species declined during the epizootic but persist to date at low abundances (e.g. *E. prosoblepon*, *P. cruentus*; electronic supplementary material, table S4, [14]).

### (e) Species contributions to community-level disease dynamics

**Figure 3.**
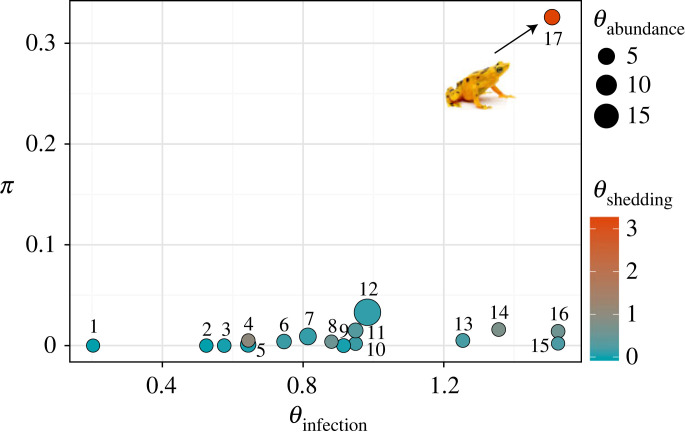
Asymmetries in host competence (based on host abundance, infection probability and pathogen shedding) determine *π*, the total contribution of host species to pathogen infection. During the outbreak, *Atelopus varius* contributed disproportionally to community disease dynamics owing to its super-abundant, super-infected and super-shedding status (*θ* > 1 for all three asymmetries). Numbers identifying species are available in the electronic supplementary material, table S5. Photo courtesy of Brian Gratwicke. (Online version in colour.)

Species-level differences in prevalence, abundance and infectiousness determined their role in community-wide infection dynamics. This partitioning analysis identified *Atelopus varius* as a super-abundant species (*θ^A^* = 3.18), with high prevalence (*θ^I^* = 1.51), that shed more zoospores (*θ^S^* = 3.33) when compared to all other members of the community (figure 3*a*; electronic supplementary material, table S5). This combination of traits distinguished *A. varius* as the key host (defined as the species with highest *π* value = 0.33) during the epizootic [8]. Four to six years post-epizootic and after the disappearance of *A. varius*, the identity of key hosts shifted (electronic supplementary material, table S5). Sixty per cent of the species that were surveyed in 2008 exhibited decreased abundance and a lower prevalence (electronic supplementary material, tables S1 and S5) than they had in 2004, indicating community-level changes in transmission dynamics after the epizootic.

**Figure 4.**
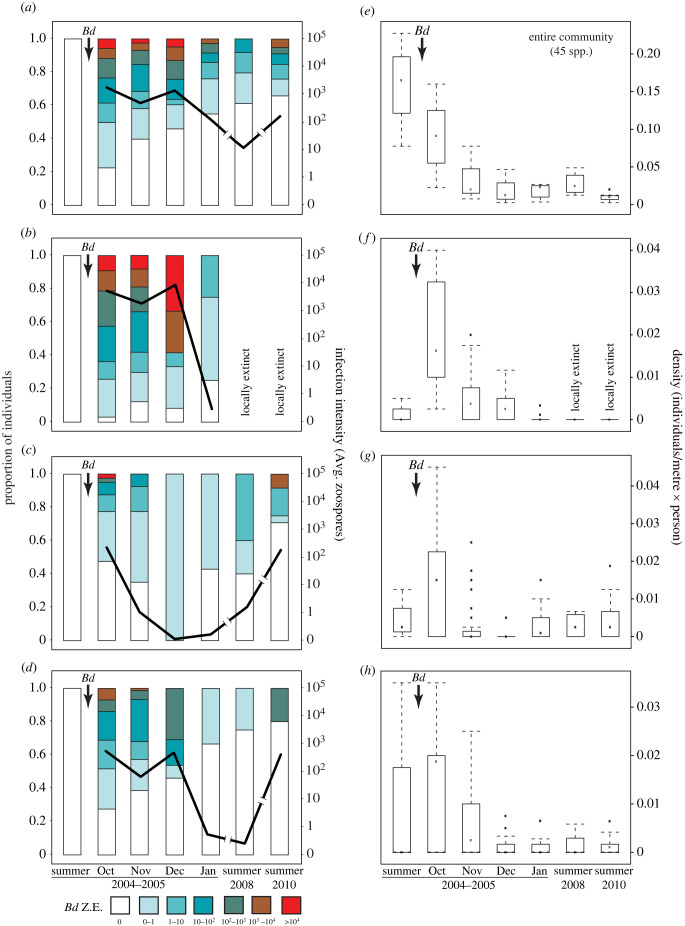
Community-level infection dynamics and individual species responses before, during and after a *Bd* epizootic at El Copé. Stacked graphs at the left (panels *a*–*d*) show the proportion of individuals infected by their fungal load. Colours represent zoospore load (number of genomic equivalents, *Bd* GE) by sampling period; the black line depicts the mean monthly load. Boxplots at the right (panels *e*–*h*) summarize the density at each time period standardized by effort for the whole community (*a*,*e*) and for the most abundant species (*Atelopus varius*: *b*,*f*; *Espadarana prosoblepon*: *c*,*g*; and *Pristimantis cruentus*: *d*,*h*).

We reconstructed the temporal infection dynamics during and after the outbreak for the entire community and for three most common species (*A. varius, E. prosoblepon, P. cruentus*; figure 4*a–d*). These three species had different reproductive modes, abundance, prevalence and infection intensity (electronic supplementary material, tables S1 and S5). *Atelopus varius* contributed highly to infection dynamics; at the height of the epizootic, 97% of *A. varius* carried high *Bd* infections (5859 ± 23 259 GE), suffered high mortality, and became extirpated by 2005 (figure 4*a*,*e*). By contrast, fewer individuals of *E. prosoblepon* and *P. cruentus* were infected (48% and 58%, respectively) during the outbreak and they carried significantly lower *Bd* loads (139 ± 1417 GE and 119 ± 678 GE, respectively; figure 4*c*,*d*; (Kruskal Wallis test: *H*_2_ = 108.9, *p* < 0.001)). All three species declined after *Bd* arrival (figure 4*e*–*h*), as did most species in the community. Four to six years after the epizootic, the average *Bd* infection intensity in the community decreased to less than 100 GE (electronic supplementary material, table S1), and abundances of surviving species did not recover to pre-epizootic levels (figure 4*e*–*h*).

## 4. Discussion

We assessed the role of ecological and evolutionary traits in predicting persistence to chytridiomycosis by comparing model predictions to actual responses of those species over the course of a fungal epizootic. The pre-epizootic community consisted of species with a mix of survival probabilities, abundances and infection susceptibility. Following the epizootic, host density, host species richness, and disease responses (prevalence and infection intensity) declined (figures 1 and 4; electronic supplementary material, table S1). The post-epizootic community was comprised species with higher survival probability, and thus lowered the average of prevalence and infection intensity in the community (figures 3 and 4). Our findings highlight the importance of integrating the concept of host competence at the community level.

### (a) Reduction in infectivity after the epizootic

Our data confirmed that *Bd* is a fungal pathogen with a broad host range, even during its first emergence in a new composition of hosts. We found that more than 80% of the species got infected with *Bd*, and those uninfected probably represented missed infections owing to small sample size. During the epizootic, we observed a reduction in species contributions to community disease dynamics (figure 4; electronic supplementary material, table S5), with more than half of the species exhibiting reductions in abundance, infection prevalence and infection intensity (electronic supplementary material, tables S1 and S5). This pattern could result from selection processes operating on hosts, on the pathogen or both [26,47].

Of all potential host species, *Bd* had higher success infecting and establishing in aquatic breeders (figures 1 and 2), possibly because aquatic habitats allow rapid pathogen dispersal and reproduction, and enhance spatial proximity of the hosts. After susceptible species disappeared, hosts persisting with reduced infection levels potentially developed an adaptive immune response. Immune resistance in frogs is achieved by mounting T-cell mediated responses to infection [48–50], by innate factors such as antimicrobial peptides [51] or combinations of these strategies. However, the efficacy of resistance to chytridiomycosis varies among species [52], depending on their ability to escape the immunosuppressing activity of the fungus [48]. The evolution of resistance effectively decreases pathogen fitness, potentially leading to antagonistic coevolution [53,54]. If the evolution of resistance in hosts at El Copé caused the observed reduction in *Bd* prevalence at the community level following the epizootic, either resistance is not complete, or the pathogen has evolved in response, because the El Copé community has not recovered demographically (figure 4). In addition, the shift in community composition and absence of amplifiers of infection contributed to reduce infection in the community. Lack of demographic recovery is a widespread pattern at Neotropical sites after *Bd* invasion [35,55–57], indicating that host population growth remains constrained by infection under enzootic conditions.

### (b) Identification of superspreaders and indicator species

Species at El Copé differed in relative abundance, prevalence and infection intensity during the epizootic. As a result, species such as *A. varius* which had high abundance, prevalence and infection intensity played a key role in disease dynamics during the epizootic (figures 3 and 4). Our data confirm results from laboratory studies showing that this species produces some of the highest numbers of zoospores in experimental infections and is thus an ‘acute supershedder’ [46,58,59]. *Atelopus* contributed to the severity of the decline at El Copé, and other species in this genus or with similar extreme competence may be key to understanding widespread declines of amphibians across the Americas.

Harlequin frogs (genus *Atelopus*) were once common members of many Central and South American montane amphibian faunas and had a wide distribution ranging from Costa Rica to Bolivia and the Guiana Shield [56]. Most species of *Atelopus* occupy riparian microhabitats in mid-elevation forested sites [56], and have an aquatic reproductive mode, two traits significantly correlated with the probability of decline and with disease severity [12]. At last count, 81% of *Atelopus* species, for which we have adequate demographic data, have either declined or disappeared owing to *Bd* emergence [28,56]. Until now, we lacked field evidence to show the level at which *Bd* could exploit *Atelopus* as a resource for rapid disease spread. Species such as *Colostethus panamansis*, *Strabomantis bufoniformis* and *Lithobathes warszewitschii* are other examples of hosts that probably played an important role in early disease dynamics (electronic supplementary material, tables S1 and S4), although we could not identify them as key hosts because they disappeared so rapidly. We hypothesize that species with similar disease-favouring traits, such as species of *Colostethus* and *Telmatobius* [60], may play similar roles in enhancing infection risk during *Bd* emergence in other naive communities in the Neotropics. In temperate regions where amphibian communities are comprised by fewer species and show less temporal variation than in the tropics, site factors determine the consistency in the identity of the hosts maintaining infections [61]. Thus, additional work to understand site-level attributes will be important to predict future disease outbreaks.

We found that as species were extirpated during the epidemic, infection levels changed over time, and species important during the epizootic were replaced by others with lower prevalence and intensity such as *E. prosoblepon and P. cruentus*. These findings confirm recent laboratory studies that show that *E. prosoblepon* does not amplify infections, produces low numbers of zoospores [62], and has high resistance based on skin peptide activity [63].

### (c) Trait-mediated impacts during pathogen emergence

We found that both ecological and evolutionary traits contributed to disease responses. Reproductive mode was the key factor determining species disease outcome. From the ecological perspective, reproductive mode probably reflects the degree of overlap between host and pathogen habitats, and therefore correlates with species' survival [12]. From the evolutionary perspective, the phylogenetic signal highlighted the ability of the pathogen to infect species with this shared trait, underscoring the importance of examining traits to improve our predictions about species persistence after disease emergence. Our analyses further identified aquatic breeders as carriers of higher infections (figure 2). Indeed, experimental inoculations and field studies have found higher infection prevalence and intensity for species with higher affinity for aquatic habitats [12,64,65]. However, none of these studies include the response of a *Bd*-naive community, but rather test habitat correlates of amphibians persisting under enzootic conditions. We demonstrate with field data that *Bd* was highly aggregated among suitable hosts [66], and that these differences in host traits probably determine species fate after disease emergence. These findings are consistent with previous reviews that pinpoint small geographical ranges, low reproductive output and aquatic habitats as factors promoting extinctions owing to climate change, habitat modification, disease or other drivers [12,67–69].

Evolutionary history of hosts also contributed to community disease response. We found similar infection intensities in closely related species, confirming that infection success (i.e. ability to proliferate within a host species) decreased with phylogenetic distance between hosts (figure 2; electronic supplementary material, table S3). Nonetheless, we found no phylogenetic correlation with estimated or observed survival probability. Phylogenetic clades encompassed multiple traits related to pathogen exposure and host defences, and thus, it is not surprising that we found discordant phylogenetic signals for different disease responses. For instance, the lack of phylogenetic signal in disease prevalence among terrestrial breeders probably resulted from terrestrial species that live in stream habitats, and lay direct-developing eggs in or near stream beds (e.g. *S. bufoniformis, C. punctariolus, C. evanesco*), resulting in higher than expected exposure (electronic supplementary material, table S1). Likewise, arboreal species with aquatic larvae, such as those in family Centrolenidae (e.g. *Espadarana prosoblepon*), carried the lowest prevalence levels and infection intensities of the aquatic breeders, indicating that habitat use might be driving the significance of reproductive mode. Therefore, a phylogenetic signal in disease responses might occur in some cases, but it will depend on the distribution and evolutionary variability of specific traits in the species contained in different clades.

### (d) How can community-level disease dynamics inform disease mitigation efforts?

Understanding phylogenetic signal in disease responses and the consequences of species-specific traits for community level disease dynamics is important for applied efforts in disease ecology. First, our results can be applied to predict which species are highly susceptible to *Bd* given their phylogenetic placement, contributing to assessments of species suitability for reintroduction programmes, or to measure the probability of moving pathogens through trade. Second, determining species persisting in assemblages after epizootics will help identify closely related species with similar traits that potentially also tolerate high infection intensities. Studies in temperate regions have shown the importance of considering community-level contributions of hosts to infection [61]. Third, experimental manipulations of species that show reproductive plasticity [70] can provide a fine-scale intraspecific model to test the evolutionary advantage of switching environments (aquatic to terrestrial) and its impacts on pathogen exposure.

Finally, our results demonstrate that enzootic disease dynamics continue to change with new species additions or losses. For example, reintroducing taxa with high susceptibility traits, without selecting for resistance, might favour new epizootics, risking other species in the community. Long-term disease surveillance and diagnostic data, such as those we employed here, provide real estimates of trait-based asymmetries in host susceptibility [8,27], and how these species contributions change through time, increasing our capacity to develop effective management interventions [71].

## 5. Conclusion

The trait-based approach we used here is applicable to any multi-host pathogens such as salamander-killing fungus, snake fungal disease, West Nile virus in birds, and white nose syndrome in bats. The strength of this approach is in defining links among host ecological traits, evolutionary history and community composition to predict the effects of disease on communities. Therefore, knowing before pathogen arrival whether the most common and abundant species of the assemblage are highly susceptible can directly benefit disease control strategies. Likewise, our community-level analyses of species traits contributing to disease are critical for identifying appropriate species and densities for *in situ* management, restoration efforts, as well as for potential control of key host density.

## Data Availability

R code and data to reproduce figures and analyses are available from the Github repository: https://github.com/anavlongo/evoeco_hostcompetence. Data also provided as electronic supplementary material [72].
